# *N*-Phenyl­piperidine-1-carbothio­amide

**DOI:** 10.1107/S1600536808025142

**Published:** 2008-08-13

**Authors:** Yu-Feng Li, Fang-Fang Jian

**Affiliations:** a Microscale Science Institute, Weifang University, Weifang 261061, People’s Republic of China

## Abstract

The title compound, C_12_H_16_N_2_S, was prepared by the reaction of with phenyl isothio­cyanate and piperidine. In the crystal structure, the mol­ecule exhibits inter­molecular N—H⋯S hydrogen bonds and weak intra­molecular C—H⋯S and C—H⋯N hydrogen-bonding inter­actions.

## Related literature

For related literature, see: Casas *et al.* (2002 ▶); Cowley *et al.* (2002 ▶); Toshiaki *et al.* (2003 ▶).
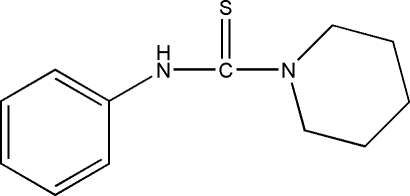


## Experimental

### 

#### Crystal data

C_12_H_16_N_2_S
*M* *_r_* = 220.33
Monoclinic, 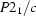

*a* = 11.661 (2) Å
*b* = 9.5220 (19) Å
*c* = 10.989 (2) Å
β = 102.15 (3)°
*V* = 1192.8 (4) Å^3^
*Z* = 4
Mo *K*α radiation
μ = 0.24 mm^−1^
*T* = 293 (2) K
0.25 × 0.20 × 0.18 mm

#### Data collection

Enraf–Nonius CAD-4 diffractometer
Absorption correction: none
2681 measured reflections
2547 independent reflections
1972 reflections with *I* > 2σ(*I*)
*R*_int_ = 0.009
3 standard reflections every 100 reflections intensity decay: none

#### Refinement

*R*[*F* ^2^ > 2σ(*F* ^2^)] = 0.040
*wR*(*F* ^2^) = 0.123
*S* = 1.02
2547 reflections
149 parameters
H atoms treated by a mixture of independent and constrained refinement
Δρ_max_ = 0.26 e Å^−3^
Δρ_min_ = −0.38 e Å^−3^

### 

Data collection: *CAD-4 Software* (Enraf–Nonius, 1989 ▶); cell refinement: *CAD-4 Software*; data reduction: *NRCVAX* (Gabe *et al.*, 1989 ▶); program(s) used to solve structure: *SHELXS97* (Sheldrick, 2008 ▶); program(s) used to refine structure: *SHELXL97* (Sheldrick, 2008 ▶); molecular graphics: *SHELXTL* (Sheldrick, 2008 ▶); software used to prepare material for publication: *WinGX* (Farrugia, 1999 ▶).

## Supplementary Material

Crystal structure: contains datablocks global, I. DOI: 10.1107/S1600536808025142/at2604sup1.cif

Structure factors: contains datablocks I. DOI: 10.1107/S1600536808025142/at2604Isup2.hkl

Additional supplementary materials:  crystallographic information; 3D view; checkCIF report

## Figures and Tables

**Table 1:** Hydrogen-bond geometry (Å, °)

*D*—H⋯*A*	*D*—H	H⋯*A*	*D*⋯*A*	*D*—H⋯*A*
N2—H2⋯S1^i^	0.82 (2)	2.78 (2)	3.5520 (19)	156.1 (18)
C1—H1*B*⋯S1	0.97	2.54	3.073 (2)	114
C5—H5*A*⋯N2	0.92 (2)	2.44 (2)	2.800 (2)	103.8 (14)

Symmetry code: (i) 
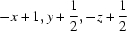
.

**Fig. 1.**
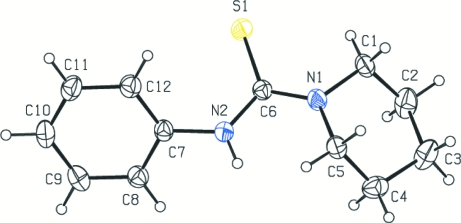
The structure of the title compound showing 30% probability displacement ellipsoids and the atom-numbering scheme.

**Fig. 2.**
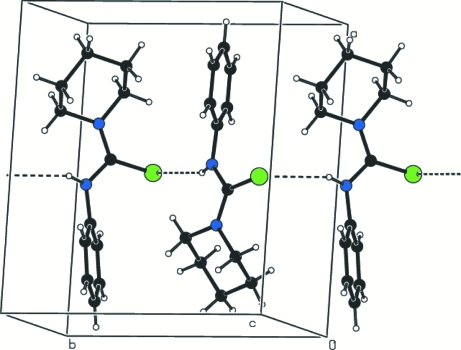
A view of the intermolecular N—H···S hydrogen bonds between the neighbour molecules in the unit cell.

## supplementary crystallographic information

### Comment

Thioamide have received considerable attention in the literature. They are attractive from several points of view in application (Toshiaki *et al.*, 2003). As part of our search for new thioamide compounds we synthesized the title compound (I), and describe its structure here.

The C6—S1 bond length of 1.7056 (17)Å is comparable with C—S bond [1.688 (2) Å] reported (Cowley *et al.*, 2002). The distance of N1—C6 [1.349 (2) Å] is similar to the distance of reported [1.349 (1) Å] (Casas *et al.*, 2002). The crystal strucure is stabilized by an intermolecular N—H···S hydrogen bond, and weak intramolecular C—H···S and C—H···N hydrogen bonding interactions (Table 1). Fig. 2 shows the intermolecular N—H···S hydrogen bonds between the neighbour molecules in the unit cell.

### Experimental

A mixture of the phenyl isothiocyanate (0.1 mol), and piperidine (0.1 mol) was stirred in refluxing ethanol (20 mL) for 4 h to afford the title compound (0.086 mol, yield 86%). Single crystals suitable for X-ray measurements were obtained by recrystallization from ethanol at room temperature.

### Refinement

H atoms were fixed geometrically and allowed to ride on their attached atoms, with C—H distances = 0.93 - 0.97 Å, and with *U*_iso_=1.2 or 1.5*U*_eq_.

### Crystal data

**Table:**

C_12_H_16_N_2_S	*F*_000_ = 472
*M**_r_* = 220.33	*D*_x_ = 1.227 Mg m^−^^3^
Monoclinic, *P*2_1_/*c*	Mo *K*α radiation λ = 0.71073 Å
Hall symbol: -P 2ybc	Cell parameters from 25 reflections
*a* = 11.661 (2) Å	θ = 1.8–27.0º
*b* = 9.5220 (19) Å	µ = 0.24 mm^−^^1^
*c* = 10.989 (2) Å	*T* = 293 (2) K
β = 102.15 (3)º	Block, colourless
*V* = 1192.8 (4) Å^3^	0.25 × 0.20 × 0.18 mm
*Z* = 4	

### Data collection

**Table:**

Enraf–Nonius CAD-4 diffractometer	*R*_int_ = 0.009
Radiation source: fine-focus sealed tube	θ_max_ = 27.0º
Monochromator: graphite	θ_min_ = 1.8º
*T* = 293(2) K	*h* = −13→13
ω scans	*k* = −11→0
Absorption correction: none	*l* = 0→13
2681 measured reflections	3 standard reflections
2547 independent reflections	every 100 reflections
1972 reflections with *I* > 2σ(*I*)	intensity decay: none

### Refinement

**Table:**

Refinement on *F*^2^	Hydrogen site location: inferred from neighbouring sites
Least-squares matrix: full	H atoms treated by a mixture of independent and constrained refinement
*R*[*F*^2^ > 2σ(*F*^2^)] = 0.040	*w* = 1/[σ^2^(*F*_o_^2^) + (0.0738*P*)^2^ + 0.2418*P*] where *P* = (*F*_o_^2^ + 2*F*_c_^2^)/3
*wR*(*F*^2^) = 0.123	(Δ/σ)_max_ < 0.001
*S* = 1.03	Δρ_max_ = 0.26 e Å^−^^3^
2547 reflections	Δρ_min_ = −0.38 e Å^−^^3^
149 parameters	Extinction correction: SHELXL97 (Sheldrick, 2008), Fc^*^=kFc[1+0.001xFc^2^λ^3^/sin(2θ)]^-1/4^
Primary atom site location: structure-invariant direct methods	Extinction coefficient: 0.129 (8)
Secondary atom site location: difference Fourier map	

### Special details

**Table:**

Geometry. All e.s.d.'s (except the e.s.d. in the dihedral angle between two l.s. planes) are estimated using the full covariance matrix. The cell e.s.d.'s are taken into account individually in the estimation of e.s.d.'s in distances, angles and torsion angles; correlations between e.s.d.'s in cell parameters are only used when they are defined by crystal symmetry. An approximate (isotropic) treatment of cell e.s.d.'s is used for estimating e.s.d.'s involving l.s. planes.
Refinement. Refinement of *F*^2^ against ALL reflections. The weighted *R*-factor *wR* and goodness of fit *S* are based on *F*^2^, conventional *R*-factors *R* are based on *F*, with *F* set to zero for negative *F*^2^. The threshold expression of *F*^2^ > σ(*F*^2^) is used only for calculating *R*-factors(gt) *etc*. and is not relevant to the choice of reflections for refinement. *R*-factors based on *F*^2^ are statistically about twice as large as those based on *F*, and *R*- factors based on ALL data will be even larger.

### Fractional atomic coordinates and isotropic or equivalent isotropic displacement parameters (Å^2^)

**Table:**

	*x*	*y*	*z*	*U*_iso_*/*U*_eq_	
S1	0.48254 (4)	0.19137 (5)	0.42566 (4)	0.04595 (19)	
N1	0.33547 (12)	0.37786 (15)	0.29140 (15)	0.0441 (4)	
N2	0.53031 (12)	0.41101 (18)	0.29001 (15)	0.0465 (4)	
C1	0.23443 (15)	0.2923 (2)	0.3048 (2)	0.0507 (5)	
H1A	0.1878	0.3431	0.3537	0.061*	
H1B	0.2615	0.2060	0.3484	0.061*	
C2	0.15982 (19)	0.2580 (2)	0.1783 (2)	0.0655 (6)	
H2B	0.0900	0.2083	0.1890	0.079*	
H2C	0.2034	0.1966	0.1341	0.079*	
C3	0.1237 (2)	0.3906 (2)	0.1007 (3)	0.0701 (7)	
H3A	0.0837	0.3642	0.0173	0.084*	
H3B	0.0696	0.4450	0.1377	0.084*	
C4	0.23033 (19)	0.4800 (2)	0.09364 (19)	0.0558 (5)	
H4A	0.2794	0.4308	0.0465	0.067*	
H4B	0.2051	0.5676	0.0512	0.067*	
C5	0.29967 (16)	0.51043 (19)	0.22297 (19)	0.0449 (4)	
C6	0.44703 (14)	0.33344 (17)	0.33069 (15)	0.0362 (4)	
C7	0.65404 (14)	0.39270 (17)	0.32415 (16)	0.0388 (4)	
C8	0.71647 (15)	0.38347 (18)	0.23028 (18)	0.0439 (4)	
H8A	0.6772	0.3841	0.1473	0.053*	
C9	0.83783 (16)	0.3733 (2)	0.2604 (2)	0.0522 (5)	
H9A	0.8797	0.3673	0.1973	0.063*	
C10	0.89689 (16)	0.3721 (2)	0.3832 (2)	0.0538 (5)	
H10A	0.9782	0.3646	0.4030	0.065*	
C11	0.83420 (16)	0.3822 (2)	0.4765 (2)	0.0533 (5)	
H11A	0.8737	0.3814	0.5594	0.064*	
C12	0.71314 (16)	0.3935 (2)	0.44777 (18)	0.0481 (4)	
H12A	0.6716	0.4015	0.5110	0.058*	
H5A	0.3633 (19)	0.566 (2)	0.220 (2)	0.055 (6)*	
H5B	0.2457 (19)	0.561 (2)	0.267 (2)	0.056 (6)*	
H2	0.5075 (17)	0.463 (2)	0.230 (2)	0.051 (6)*	

### Atomic displacement parameters (Å^2^)

**Table:**

	*U*^11^	*U*^22^	*U*^33^	*U*^12^	*U*^13^	*U*^23^
S1	0.0424 (3)	0.0424 (3)	0.0520 (3)	0.00420 (18)	0.00770 (19)	0.01026 (19)
N1	0.0343 (7)	0.0404 (8)	0.0576 (9)	−0.0006 (6)	0.0095 (6)	0.0122 (7)
N2	0.0336 (7)	0.0551 (9)	0.0494 (9)	−0.0004 (7)	0.0058 (6)	0.0162 (7)
C1	0.0344 (9)	0.0481 (10)	0.0717 (13)	0.0002 (7)	0.0158 (8)	0.0176 (9)
C2	0.0502 (11)	0.0419 (11)	0.0962 (17)	−0.0057 (9)	−0.0033 (11)	0.0036 (11)
C3	0.0613 (13)	0.0556 (13)	0.0792 (16)	−0.0016 (10)	−0.0179 (11)	0.0042 (11)
C4	0.0655 (12)	0.0503 (11)	0.0525 (11)	0.0128 (9)	0.0142 (9)	0.0097 (9)
C5	0.0370 (8)	0.0355 (9)	0.0631 (12)	0.0021 (7)	0.0124 (8)	0.0092 (8)
C6	0.0351 (8)	0.0369 (8)	0.0367 (8)	−0.0014 (6)	0.0078 (6)	−0.0016 (6)
C7	0.0335 (8)	0.0343 (8)	0.0475 (9)	−0.0029 (6)	0.0059 (7)	0.0017 (7)
C8	0.0418 (9)	0.0420 (10)	0.0474 (10)	−0.0041 (7)	0.0081 (7)	0.0007 (7)
C9	0.0434 (10)	0.0475 (11)	0.0706 (13)	−0.0022 (8)	0.0233 (9)	−0.0035 (9)
C10	0.0320 (9)	0.0451 (10)	0.0814 (14)	−0.0008 (7)	0.0052 (9)	0.0032 (9)
C11	0.0435 (10)	0.0539 (12)	0.0556 (11)	−0.0051 (8)	−0.0054 (8)	0.0008 (9)
C12	0.0428 (9)	0.0536 (11)	0.0466 (10)	−0.0036 (8)	0.0066 (8)	−0.0027 (8)

### Geometric parameters (Å, °)

**Table:**

S1—C6	1.7056 (17)	C4—C5	1.508 (3)
N1—C6	1.349 (2)	C4—H4A	0.9700
N1—C1	1.465 (2)	C4—H4B	0.9700
N1—C5	1.484 (2)	C5—H5A	0.92 (2)
N2—C6	1.368 (2)	C5—H5B	0.99 (2)
N2—C7	1.423 (2)	C7—C8	1.385 (3)
N2—H2	0.82 (2)	C7—C12	1.388 (2)
C1—C2	1.512 (3)	C8—C9	1.387 (3)
C1—H1A	0.9700	C8—H8A	0.9300
C1—H1B	0.9700	C9—C10	1.380 (3)
C2—C3	1.533 (3)	C9—H9A	0.9300
C2—H2B	0.9700	C10—C11	1.383 (3)
C2—H2C	0.9700	C10—H10A	0.9300
C3—C4	1.522 (3)	C11—C12	1.384 (3)
C3—H3A	0.9700	C11—H11A	0.9300
C3—H3B	0.9700	C12—H12A	0.9300
			
C6—N1—C1	122.35 (14)	H4A—C4—H4B	108.2
C6—N1—C5	125.33 (14)	N1—C5—C4	110.63 (16)
C1—N1—C5	112.18 (14)	N1—C5—H5A	111.5 (13)
C6—N2—C7	126.72 (15)	C4—C5—H5A	110.9 (13)
C6—N2—H2	117.1 (14)	N1—C5—H5B	107.9 (13)
C7—N2—H2	115.0 (14)	C4—C5—H5B	106.3 (12)
N1—C1—C2	110.30 (17)	H5A—C5—H5B	109.3 (18)
N1—C1—H1A	109.6	N1—C6—N2	115.41 (15)
C2—C1—H1A	109.6	N1—C6—S1	122.54 (12)
N1—C1—H1B	109.6	N2—C6—S1	122.05 (12)
C2—C1—H1B	109.6	C8—C7—C12	119.93 (16)
H1A—C1—H1B	108.1	C8—C7—N2	118.31 (16)
C1—C2—C3	111.80 (18)	C12—C7—N2	121.62 (16)
C1—C2—H2B	109.3	C7—C8—C9	119.78 (18)
C3—C2—H2B	109.3	C7—C8—H8A	120.1
C1—C2—H2C	109.3	C9—C8—H8A	120.1
C3—C2—H2C	109.3	C10—C9—C8	120.50 (19)
H2B—C2—H2C	107.9	C10—C9—H9A	119.7
C4—C3—C2	110.95 (18)	C8—C9—H9A	119.7
C4—C3—H3A	109.4	C9—C10—C11	119.48 (17)
C2—C3—H3A	109.4	C9—C10—H10A	120.3
C4—C3—H3B	109.4	C11—C10—H10A	120.3
C2—C3—H3B	109.4	C10—C11—C12	120.59 (18)
H3A—C3—H3B	108.0	C10—C11—H11A	119.7
C5—C4—C3	109.95 (18)	C12—C11—H11A	119.7
C5—C4—H4A	109.7	C11—C12—C7	119.70 (18)
C3—C4—H4A	109.7	C11—C12—H12A	120.1
C5—C4—H4B	109.7	C7—C12—H12A	120.1
C3—C4—H4B	109.7		
			
C6—N1—C1—C2	−117.8 (2)	C7—N2—C6—N1	175.49 (17)
C5—N1—C1—C2	58.1 (2)	C7—N2—C6—S1	−3.9 (3)
N1—C1—C2—C3	−54.1 (2)	C6—N2—C7—C8	129.22 (19)
C1—C2—C3—C4	52.7 (3)	C6—N2—C7—C12	−55.1 (3)
C2—C3—C4—C5	−53.8 (3)	C12—C7—C8—C9	0.8 (3)
C6—N1—C5—C4	115.4 (2)	N2—C7—C8—C9	176.49 (16)
C1—N1—C5—C4	−60.4 (2)	C7—C8—C9—C10	0.1 (3)
C3—C4—C5—N1	57.2 (2)	C8—C9—C10—C11	−0.5 (3)
C1—N1—C6—N2	167.41 (17)	C9—C10—C11—C12	0.0 (3)
C5—N1—C6—N2	−8.0 (3)	C10—C11—C12—C7	0.9 (3)
C1—N1—C6—S1	−13.2 (2)	C8—C7—C12—C11	−1.3 (3)
C5—N1—C6—S1	171.48 (14)	N2—C7—C12—C11	−176.82 (17)

### Hydrogen-bond geometry (Å, °)

**Table:**

*D*—H···*A*	*D*—H	H···*A*	*D*···*A*	*D*—H···*A*
N2—H2···S1^i^	0.82 (2)	2.78 (2)	3.5520 (19)	156.1 (18)
C1—H1B···S1	0.97	2.54	3.073 (2)	114
C5—H5A···N2	0.92 (2)	2.44 (2)	2.800 (2)	103.8 (14)

Symmetry codes: (i) −*x*+1, *y*+1/2, −*z*+1/2.
